# Systematic comparison of ranking aggregation methods for gene lists in experimental results

**DOI:** 10.1093/bioinformatics/btac621

**Published:** 2022-09-12

**Authors:** Bo Wang, Andy Law, Tim Regan, Nicholas Parkinson, Joby Cole, Clark D Russell, David H Dockrell, Michael U Gutmann, J Kenneth Baillie

**Affiliations:** Roslin Institute, University of Edinburgh, Edinburgh EH25 9RG, UK; Roslin Institute, University of Edinburgh, Edinburgh EH25 9RG, UK; Roslin Institute, University of Edinburgh, Edinburgh EH25 9RG, UK; Roslin Institute, University of Edinburgh, Edinburgh EH25 9RG, UK; University of Sheffield, Sheffield S10 2NT, UK; Centre for Inflammation Research, The Queen’s Medical Research Institute, University of Edinburgh, Edinburgh EH16 4TJ, UK; Centre for Inflammation Research, The Queen’s Medical Research Institute, University of Edinburgh, Edinburgh EH16 4TJ, UK; School of Informatics, University of Edinburgh, Edinburgh EH8 9AB, UK; Roslin Institute, University of Edinburgh, Edinburgh EH25 9RG, UK

## Abstract

**Motivation:**

A common experimental output in biomedical science is a list of genes implicated in a given biological process or disease. The gene lists resulting from a group of studies answering the same, or similar, questions can be combined by ranking aggregation methods to find a consensus or a more reliable answer. Evaluating a ranking aggregation method on a specific type of data before using it is required to support the reliability since the property of a dataset can influence the performance of an algorithm. Such evaluation on gene lists is usually based on a simulated database because of the lack of a known truth for real data. However, simulated datasets tend to be too small compared to experimental data and neglect key features, including heterogeneity of quality, relevance and the inclusion of unranked lists.

**Results:**

In this study, a group of existing methods and their variations that are suitable for meta-analysis of gene lists are compared using simulated and real data. Simulated data were used to explore the performance of the aggregation methods as a function of emulating the common scenarios of real genomic data, with various heterogeneity of quality, noise level and a mix of unranked and ranked data using 20 000 possible entities. In addition to the evaluation with simulated data, a comparison using real genomic data on the SARS-CoV-2 virus, cancer (non-small cell lung cancer) and bacteria (macrophage apoptosis) was performed. We summarize the results of our evaluation in a simple flowchart to select a ranking aggregation method, and in an automated implementation using the meta-analysis by information content algorithm to infer heterogeneity of data quality across input datasets.

**Availability and implementation:**

The code for simulated data generation and running edited version of algorithms: https://github.com/baillielab/comparison_of_RA_methods. Code to perform an optimal selection of methods based on the results of this review, using the MAIC algorithm to infer the characteristics of an input dataset, can be downloaded here: https://github.com/baillielab/maic. An online service for running MAIC: https://baillielab.net/maic.

**Supplementary information:**

Supplementary data are available at *Bioinformatics* online.

## 1 Introduction

In biology, there are usually many results from different sources for the same or a similar problem. Many results take the form of a list of genes or proteins, especially for screens of genes, transcripts and proteins related to a specific biological process. In almost all cases, these gene lists overlap with results from other experiments. Meta-analysis aims to combine the individual gene lists resulting from individual studies to obtain a more reliable answer. Meta-analysis of this kind of data can often be seen as a ranking aggregation and there are many methods for carrying it out (Li *et al.*, 2019).

There are various existing methods for ranking aggregation. This study focuses on the unsupervised and rank-based methods since for transcriptomic and genomic level data there are usually no high-quality training datasets with reliable target labels or universally accepted methods for quantification across different data sources (Li *et al.*, 2019; Liu *et al.*, 2007).

Unranked lists are common in biology. Examples include annotated pathways [e.g. KEGG (Kanehisa *et al.*, 2019), Reactome (Jassal *et al.*, 2020), Wikipathways (Slenter *et al.*, 2018)], co-expression clusters [e.g. FANTOM5 (Andersson *et al.*, 2014) and STRING database (Szklarczyk *et al.*, 2016)] and reports providing a group of entities as the positive result of a study without ranking them. However, unranked lists are not explicitly accommodated by many aggregation methods and are often either excluded from meta-analyses or incorporated in an *ad hoc* manner.

All methods investigated in this study can deal with ranked lists as input (reported genes with order information), irrespective of whether each list includes all possible entities or not. However, only a few methods among them can accept unranked lists as input. Some approaches like meta-analysis by information content (MAIC) (Li *et al.*, 2020) and VoteCounting (Li *et al.*, 2020) explicitly claim that they can deal with unranked data. Some methods which are designed only for ranked lists can also tackle this type of input with a slight change to the algorithm, such as Borda’s methods (Brancotte *et al.*, 2015). An unranked source can be a special case of ranking with ties (entities with the same ranking) (Brancotte *et al.*, 2015) so that methods that can deal with ranking with ties can also accept unranked sources. An example can be RepeatChoice (Ailon, 2010), which breaks ties starting from an input ranking using order information of other sources. A study (Brancotte *et al.*, 2015) on ranking with ties modified some methods to adapt them to ties, like Borda’s methods. Borda’s methods have variations using statistics like mean value (MEAN, GEO) or median (MED) (de Borda, 1781; Lin, 2010) and can accept an unranked list by assigning the same ranking to entities within the list, which is intuitively reasonable for unranked sources. In contrast, unranked sources are not explicitly accommodated by some relatively complicated methods although ideas about dealing with absent ranking information especially for ranked top-d partial lists are sometimes proposed. Examples can be Bayesian methods like BiG (Li *et al.*, 2018) and BARD (Deng *et al.*, 2014), with a solid Bayesian theoretical framework and an estimated distribution instead of only a ranking as the result for each algorithm.

The dataset and the problem at hand can influence the applicability of an algorithm a lot. For example, the inclusion of unranked sources can make methods that only accept ranked sources unusable. Noise level and heterogeneity of the noise are also important properties of the data sources and not every method can perform well on very noisy partial ranked data (Kolde *et al.*, 2012). Optimizing a metric that treats all sources equally in the calculation, such as the MEAN method of Borda’s methods, is not suitable when significant noise is included (Kolde *et al.*, 2012). Moreover, a large number of elements (e.g. around 20 000 genes for humans) also makes methods like cross-entropy Monte Carlo (Lin and Ding, 2009) unsuitable because of the computational cost (Li *et al.*, 2019). Considering the features of the genomic datasets, a specific evaluation of ranking aggregation methods is thus required to establish their performance.

Ranking aggregation methods usually use simulated datasets for evaluation (Deng *et al.*, 2014; Kolde *et al.*, 2012; Li *et al.*, 2019; Yi *et al.*, 2016) as there is a lack of large genomic real datasets with definitive answers. In contrast, simulated data provide ground truth values and can also be used to investigate the effect that specific properties of the real data, like the amount of noise or heterogeneity in the source quality, have on the different algorithms.

In many existing studies, including RRA (Kolde *et al.*, 2012), BARC (Yi *et al.*, 2016), BiG (Li *et al.*, 2018), BIRRA (Badgeley *et al.*, 2015) and MAIC (Li *et al.*, 2020), simulated data were used. Existing research explored datasets with some features of genomic data, including various partial cases to cut lists, noise levels, heterogeneity of source quality (Li *et al.*, 2019, 2020) and the way of setting the top 5% as truth (Badgeley *et al.*, 2015; Kolde *et al.*, 2012). But some important features for genomic data, including the difference between the top-ranked genes in the truth set and a large number of entities (like over 20 000 genes for humans), have not been systematically explored. To evaluate MAIC (Li *et al.*, 2020), 500 entities were used in the simulated data, and the evaluations in Li *et al.* (2019) and Badgeley *et al.* (2015) used 1000 entities for the simulated data. Assigning a score for each entity subject to noise from some pre-defined distributions is a common way to generate simulated data. The expected mean score of each entity is an arbitrarily defined constant to show the difference in the significance of genes for the simulation. These scores rank genes from top-ranked genes to bottom or classify genes into truth group and noise group (Kolde *et al.*, 2012; Li *et al.*, 2018; Yi *et al.*, 2016).

In previous research about the MAIC algorithm (Li *et al.*, 2020), the dataset used in the evaluation is generated by ranking *Z* scores sampled from independent normal distributions given a list specified precision (inverse covariance). This data generation model can control the heterogeneity of list quality and average noise level but was in previous research only used to generate small datasets and short lists (500 potential entities) in order to compare the RRA, MAIC and vote counting (VC) method. It has a high potential to generate well-simulated data and forms the basis of the new data simulation method proposed in this study.

**Fig. 1.**
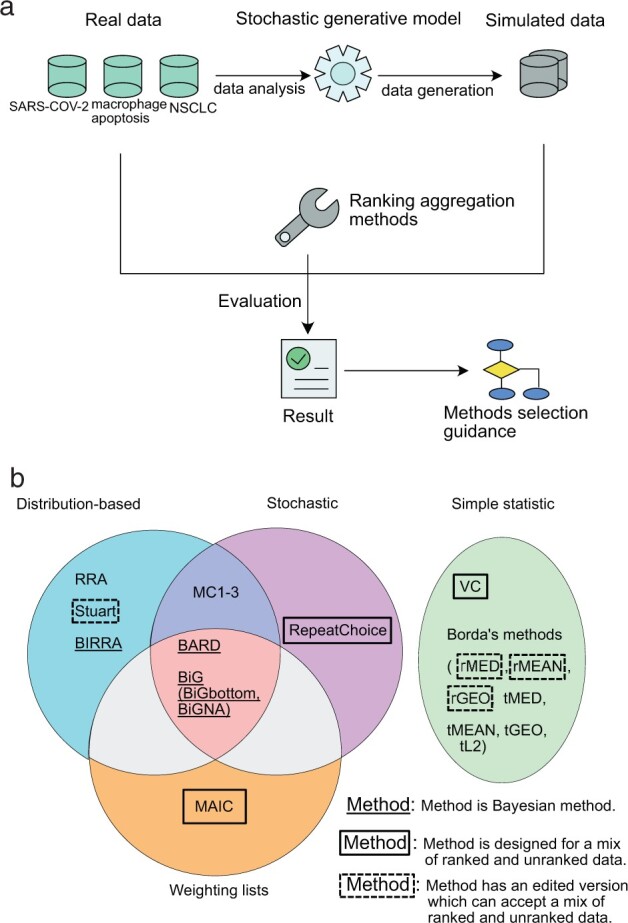
(**a**) An overview of the methods for this study. (**b**) Investigated ranking aggregation methods in this study and some key features of them. Distribution based: the method is based on the distribution of latent model or calculated statistics (Li *et al.*, 2019). Stochastic: the method includes a stochastic process like random sampling. Weighting lists: the method assigns weightings to lists explicitly to show their difference, like quantifying the quality. Simple statistics: use simple statistics, like frequency or average ranking. Bayesian methods are labeled using underline. Whether the methods are designed to only take ranked lists as the input or be able to accept unranked lists are also marked

As summarized in Figure 1a, we first generated realistic synthetic datasets to simulate real features of biological experimental results. We then used these synthetic data, together with real data from selected fields of biology, to systematically evaluate aggregation methods in a range of conditions expected to be encountered in real-world conditions (mixed versus ranked data, large dataset sizes, heterogeneity and noise).

### 1.1 Contribution

- Viral infection (SARS-CoV-2), cancer [non-small cell lung cancer (NSCLC)] and bacterial infection (modulation of macrophage apoptosis) datasets were collected. Each set used in the assessment is extracted from sources either more highly related to the research question of the corresponding meta-analysis or published in a closer time and is considered to be highly reliable.
- A new simulated data generation method is proposed by analyzing three real datasets in terms of list number, length, order information, quality of sources, heterogeneity of quality, absent genes and also the relationship between each significant gene.
- Implementation of investigated ranking aggregation methods and variations of them that are suitable for dealing with genomic data was carried out based on existing source code, enabling them to use the data with the same format easily and providing the ability of using unranked lists as input for some algorithms. They are evaluated systematically on both the real and the simulated datasets.
- The overall evaluation results are condensed in a practical flowchart to select appropriate ranking aggregation methods depending on the ranking information, the number of included sources and the heterogeneity in the quality of the data sources.

## 2 Materials and methods

### 2.1 Real data collection

Three real datasets are collected, corresponding to virus (SARS-CoV-2), bacteria (macrophages apoptosis) and cancer (NSCLC), as shown in Table 1. A detailed description of it is in Supplementary File S1—Section S1.

**Table 1:** Sources of the collected real data

Dataset	Extracted lists	Source
SARS-CoV-2	11 ranked + 21 unranked lists	Extracted lists till July 9, 2020 in Parkinson *et al.* (2020)
	Truth to assess the performance	Lists from July 9, 2020 to November 25, 2020 in Parkinson *et al.* (2020)
NSCLC	5 ranked lists	Borczuk *et al.* (2003), Kerkentzes *et al.* (2014), Li *et al.* (2015), Zhou *et al.* (2017) and Kim *et al.* (2013)
	Truth to assess the performance	Truth used in Li *et al.* (2018), provided in Li *et al.* (2019) and Chen *et al.* (2013)
Macrophage apoptosis	4 ranked lists	2 from SHIELD AMR Research Consortium (https://shieldamrresearch.org/) (Preston *et al.*, 2019) The other 2 from Huang *et al.* (2020) and Kumar *et al.* (2010)
	2 unranked lists	MacHugh *et al.* (2012) and Yeung *et al.* (2019)
	Truth to assess the performance	Abebe *et al.* (2010), Losick and Isberg (2006), Maertzdorf *et al.* (2011) and Lai *et al.* (2020)

### 2.2 Simulated data and experiment design

In order to better explore the properties of real data, which usually includes a large number of entities, a new stochastic generative model that emulates real data are proposed. It includes 20 000 potential entities (human genome scale) in total and incorporates heterogeneity in the lengths of lists. The top-1000 (5%) entities will be considered as signal/significant entities, which correspond to the number of top-ranked genes being focused on in real research.

For list *L_i_*, entity *E_k_* (*k* = 1…20 000), mean noise scale *M*, mean cutting point *m_c_*, heterogeneity of noise *D*, heterogeneity of cutting point *D_c_*, range for each list length [L,U], entity significance *μ_k_* for entity *E_k_*, the score *Z_ki_* for entity *E_k_* in list *L_i_* is

$$\begin{array}{c} Z_{ki}∼N(\mu_{k},\sigma_{i}^{2})\\ \ln(\sigma_{i})∼N(\ln(M),D) \end{array}$$

where $N(a,b^{2})$ denotes a Gaussian distribution with mean *a* and variance *b*^2^. The number of entities which will be finally included in list *L_i_* is denoted by $N_{i}\in [L,U]$ and generated as follows:

$$\begin{array}{c} N_{i}=\min(\left\lfloor C_{i}\right\rfloor +L,U)\\ \ln(C_{i})∼N(\ln(m_{c}),D_{c}^{2}) \end{array}$$

$\left\lfloor C_{i}\right\rfloor +L$ is the cutting point for list *L_i_* to only keep the top *N_i_* entities in the list and $N_{i}\in [L,U]$. $\ln(C_{i})$ is used instead of $C_{i}$ to make the perturbation on a larger scale easier than a smaller scale, because the difference on a larger scale of length is considered to be less significant. For example, the difference between length 2 and 102 are more significant than the difference between length 19 000 and 19 100. After the generation, all the potential entities within list *L_i_* are ranked by score *Z_ki_*. Then, *X* entities will be removed randomly from list *L_i_* as controlled by a ratio *γ*,

$$X=\left\lfloor \gamma *(20000-N_{i})\right\rfloor ,$$

followed by removing the bottomed ranked entities until there are only *N_i_* entities left. Then list *L_i_* is labeled as ‘RANKED’ or ‘UNRANKED’ to indicate whether the order information for these *N_i_* entities are provided or not. The settings of the hyperparameters of the model, i.e. *L*, *U*, *m_c_*, *D_c_*, *M*, *D*, *μ_k_*, *γ*, the number of the lists and whether each list is ranked or unranked, are discussed in Supplementary File S1—Section S2.

In a real study, the length of the reported list is usually a result of two factors. The first one is that bottom-ranked genes are removed and not reported, whereas the second one is that some genes are not included in the study in the first place when the study is not genome-wide. These two situations were both explored by Li *et al.* (2019) and the new proposed model outlined above. The first case is emulated by removing bottom entities for simulated lists whereas the second case is emulated by removing entities (uniformly) at random.

The details about the exploration and parameter settings are included in Supplementary File S1—Section S2. List number, order information, list length, frequency decay of top-ranked entities, list quality and absent genes of three collected real datasets were assessed and the model parameters were set such that the simulated data can well emulate the real scenarios. Depending on the number of sources in a dataset (Large or Small) and whether unranked lists are included (Mix or Rank), four groups of datasets were generated. For each of the four types of data, various values for *M*, *D*, and *γ* are used to generate different datasets that cover different scenarios of noise levels, heterogeneity and absent gene rates. A ‘Mix’ dataset has exactly the same ranked lists as the corresponding ‘Rank’ dataset and the only difference between them is the inclusion of additional unranked lists. It enables the comparison of results between datasets with and without unranked lists to explore the effects of including unranked lists for methods that can accept unranked input sources.

### 2.3 Selection and implementation of existing methods

As shown in Figure 1b, 18 methods and their typical variations are selected to include various properties of ranking aggregation methods and are suitable to deal with genomic data. They were implemented based on existing code if accessible. The implementation language for MAIC (Li *et al.*, 2020), VC and RepeatChoice is Python, whereas it is R for all other investigated methods except for BARD (Deng *et al.*, 2014), which has available code implemented in C++. RepeatChoice and VC are implemented based on the algorithms introduced in Ailon (2010) and Li *et al.* (2020), respectively, whereas other investigated methods are all implemented based on the available code. Similar to the research by Li *et al.* (2019), Borda’s methods (de Borda, 1781; Lin, 2010) are labeled with ’r’ and ’t’ to show different implementations from the RobustRankAggreg package and TopKList package. BiGbottom (treating absent genes as bottom-ranked) and BiGNA (treating the order information of absent genes as unknown) are two different versions of BiG (Li *et al.*, 2018). For Borda’s methods and Stuart algorithm (Stuart *et al.*, 2003) from R package—RobustRankAggreg, each of them can easily be edited to enable the input of unranked lists. They are edited by setting genes in unranked lists to have the same ranking, which is half of the list length. The new names for them are rMixMED, rMixMEAN, rMixGEO and MixStuart. For algorithms that are not able to deal with unranked lists, they only take ranked lists as the input for the evaluation in this study. In terms of an evaluation of a mix of ranked and unranked sources, the unranked lists are not used when they are available for these algorithms, which emulate the real usage case of these algorithms. The details of methods selection and implementation are shown in Supplementary File S1—Section S3.

## 3 Results

**Fig. 2.**
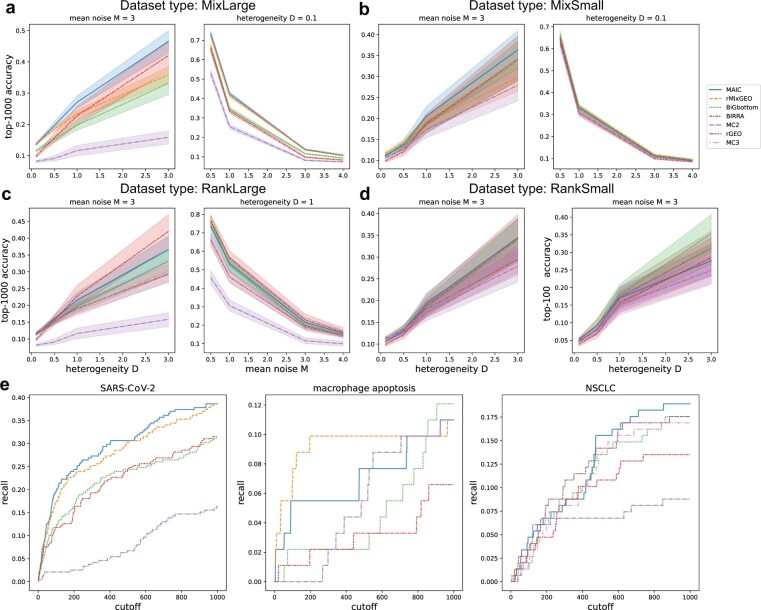
Results for simulated datasets and real datasets. All subplots use the same color and line styles to show investigated methods. Detailed results can be seen in Supplementary File S1 and Supporting data. (**a–d**) Results for simulated data with various mean noise levels and quality heterogeneity. The mean of accuracy using top-1000 cutoff (except for the right figure of d which uses top-100 cutoff) and 95% confidence interval are plotted for 100 repeated experiments using lines and shading separately. The default setting of absent gene rate *γ *= 0 is used. The simulated dataset type shows properties for datasets. The first part shows whether a dataset includes a mix of ranked and unranked sources (Mix) or only includes ranked sources (Rank). The second part shows the number of included sources (large or small). (**e**) Real data: results for the collected SARS-CoV-2, macrophages apoptosis and NSCLC datasets. The recall with cutoffs from top-1 to top-1000 is shown

This section includes the results of experiments on real and simulated data. Only the best-performing methods and some methods with interesting methodology or performance are shown in Figure 2 for important experiments to avoid clutter in the figure. Full details of the results are provided in Supplementary File S1 and Supporting data with all investigated methods included.

### 3.1 Performance measurements

The measurement used to show the accuracy of methods in gene data should be able to weight top-ranked genes more than bottom ones since they are usually more important in biological research. But, how important each position is compared with others is not known. Weighting each position can be used when comparing two lists, like average overlap (AO) (Webber *et al.*, 2010; Wu and Crestani, 2003) which provides a score with built-in importance of each position. But the result can be largely influenced by the way a measurement weights each position. To cover the various scenarios in biological research and provide easily understandable results, coverage rates for various cutoffs are selected as the measurements, similar to the measurements used in Li *et al.* (2018, 2019). Specifically, it is the accuracy value for experiments on simulated data since the ranking for truth is known. Recall value is used to measure the coverage rate in real data experiments since only unranked truth sets are available. All simulated datasets are generated with 100 repeats for each combination of parameters and 95% confidence intervals are plotted using shading in Figure 2. The result with top-1000 cutoff is mainly explored, together with a comparison with other smaller cutoffs in Supplementary File S1—Section S4 and Supporting data.

### 3.2 Result for datasets with a mix of ranked and unranked sources

It can be seen from Figure 2a that MAIC gives the best performance on a large dataset with both ranked and unranked lists like dataset type MixLarge which emulates SARS-CoV-2 dataset, especially when there is a relatively large heterogeneity of list quality (shown as *D* in simulation). It also gives the best performance for nearly all the cutoffs from top-1 to top-1000 in the evaluation of SARS-CoV-2 datasets as shown in Figure 2e. Another one of the best-performing methods for the SARS-CoV-2 dataset is rMixGEO, which reaches the best level to have similar performance as MAIC when heterogeneity is small in the simulated data, like *D *=* *0.1. As a method using simple statistics, rMixGEO wins against the most complicated methods in this scenario, but it is not as robust as MAIC when heterogeneity is high.

In terms of a relatively small dataset with a mix of ranked and unranked sources (MixSmall in the simulation as shown in Fig. 2b), MAIC and rMixGEO follow the same performance pattern as for large datasets. MAIC still reaches the highest performance level whereas rMixGEO experiences a decreasing performance ranking among the investigated methods as the heterogeneity increases. One interesting point is that the performance ranking of BIRRA is influenced a lot by both mean noise and heterogeneity, becoming top ranked when heterogeneity is high and mean noise is low for small datasets (see Supplementary File S1—Fig. S9).

For each NSCLC and macrophage apoptosis data, both input lists and the gold standard truth are extracted from less than 10 sources. So the results of NSCLC and macrophage apoptosis data are likely to be quite noisy and less informative than SARS-CoV-2 results. But as the macrophage apoptosis result shows in Figure 2e, the results of these real data still identify that some methods roughly outperform others. rMixGEO and MAIC still show a relatively good performance whereas BIRRA does not perform as well as them.

BIRRA and BiGbottom further produce top-ranked results when heterogeneity is high and mean noise is low for small simulated datasets, but they are not as robust to a large mean noise level as MAIC (see Supplementary File S1—Fig. S9). Plotted in Figure 2b and Supplementary File S1—Figure S9, top-performed methods including MAIC and rMixGEO are all robust to a change of noise levels under the heterogeneity where they outperform others (all investigated heterogeneity for MAIC and low heterogeneity for rMixGEO) and especially perform well for classic cases (*M* is 3, which is the classic case that emulates real datasets best, see Supplementary File S1—Section S2). They are also robust to various cutoffs and absent gene rates for sources in their corresponding top performed heterogeneity scenarios (all heterogeneity for MAIC and low heterogeneity for rMixGEO), shown in Supplementary File S1—Figure S6, and also the comparison between the result with top-1000 cutoff and top-100 cutoff plotted in Supplementary File S1—Section S4.

### 3.3 Result for datasets with only ranked sources

The results for large datasets with only ranked sources are shown in Figure 2c. To avoid duplicates, the rMixGEO is not shown since it is the ‘Mix’ version of rGEO and performs exactly the same as rGEO for ranked lists. In this figure, the ranking of BIRRA shows the best performance among investigated methods for large datasets with only ranked sources (RankLarge) when heterogeneity *D* reaches 1. The ranking of it tends to be robust to the mean noise level in this scenario. For smaller heterogeneity, rGEO, BiGbottom, MAIC, rMEAN (see the results of rMEAN in Supplementary File S1 and Supporting data) and MC3 are top ranked with similar performance, whereas BiGbottom and MAIC are more robust for high heterogeneity.

Small datasets with only ranked sources (RankSmall) prefer MAIC, BIRRA and BiGbottom for the high heterogeneity case like *D* is 3, showing the similar best performance for them among investigated methods for top-1000 accuracy, as shown in Figure 2d (left). Among them, the accuracy of BiGbottom is also one of the highest when heterogeneity is small (*D* equals 0.1), with a similar performance as rGEO and MC3 in this scenario. BiGbottom also shows better robustness in terms of a change of cutoffs than MAIC and BIRRA. Compared with the result with top-1000 cutoff where these three methods show similar performance in the high heterogeneity case, BiGbottom obviously outperforms other investigated methods for top-100 cutoff result, shown in Figure 2d (right). Whereas MAIC and BIRRA are outperformed by rGEO.

Similar to macrophage apoptosis data, NSCLC result also tends to be relatively less informative since the lack of sources for the input dataset and truth set. However, MAIC, BIRRA, rGEO, MC3 and BiGbottom all perform relatively well shown in the corresponding plot in Figure 2e.

## 4 Discussion

The difference between the performance of investigated ranking aggregation methods on the proposed simulated datasets and three real datasets was compared. The results show that whether to include unranked lists for input data and the heterogeneity of quality for sources can largely influence the performance of the investigated ranking aggregation methods.

The evaluation result in this study can provide some insights on the data selection and method selection for ranking aggregation of genomic problems.

Data selection: Using a mix of ranked and unranked data instead of giving up unranked sources can lead to a better result, as shown in the comparison between the results for sources with unranked sources (Fig. 2a and b) and the results for only ranked sources (Fig. 2c and d). Compared to the ‘Rank’ datasets which only use ranked sources, the ‘Mix’ datasets include unranked sources as additional input data for those methods which can use unranked lists. The ranked lists included in the corresponding ‘Mix’ and ‘Rank’ datasets are the same so that the comparison between these datasets can show the effect of using additional unranked sources for methods that can accept them. When unranked lists are included, the accuracy can grow impressively especially for those top-performing methods, which is shown by the result of MAIC and rMixGEO (rMixGEO is a variation of rGEO which can accept unranked lists as input) that accept a mix of ranked and unranked data as input. Their performance improved substantially when unranked lists are included (left figures of Fig. 2a and b) compared to using only ranked sources (left figures of Fig. 2c and d), reaching the best performance among investigated methods with appropriate heterogeneity. It shows that unranked data has useful information to improve the meta-analysis.

**Fig. 3.**
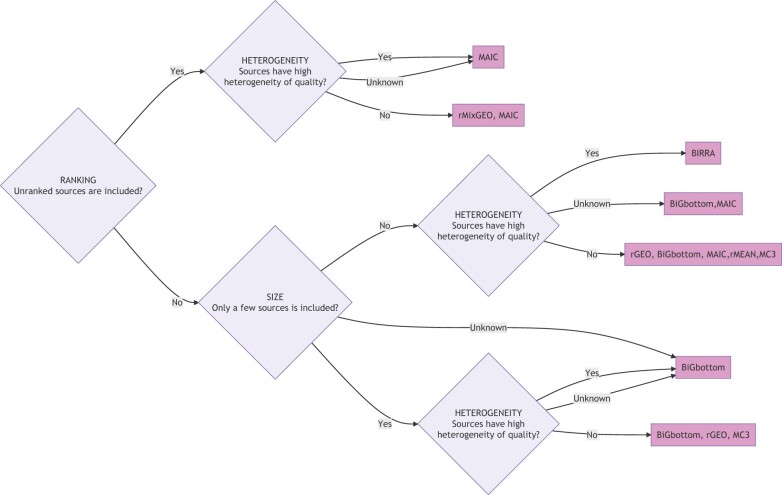
A flowchart for selecting methods depending on the ranking information, the number of sources included and the heterogeneity of quality for the investigated sources, generated following the evaluation result of this study. Multiple methods within the same block means they perform similarly with the best performance under the corresponding scenario

Method selection: Considering all the evaluation results, a flowchart for selecting methods depending on the fundamental properties of the available data is shown in Figure 3. In order to construct the flowchart, the accuracy with a top-1000 cutoff for the result of simulated datasets is firstly considered to select methods in each scenario, followed with a robust checking for various mean noise levels, cutoffs used for the measurement and absent gene rates to only select methods with relatively good robustness for these properties.

MAIC and rMixGEO show the best performance among all the methods investigated when using a mix of ranked and unranked data, while MAIC is more robust for the relatively high heterogeneity case. The method rMixGEO, which is based on simple statistics, is more intuitive and easier to implement and can be selected when the heterogeneity of quality is known to be low, such as a dataset with many sources from the same repeated experiments.

If a dataset only includes ranked data, rGEO, BiGbottom, MAIC, rMEAN, MC3 and BIRRA are preferred for a large dataset with many sources (like dataset type RankLarge). Except for rMEAN, they also show a top-ranked performance for relatively small datasets with only a few sources (like dataset type RankSmall). Among them, the ranking of BiGbottom is more robust for heterogeneity whereas BIRRA performs better for high heterogeneity scenarios than low heterogeneity scenarios among investigated methods.

In terms of the relatively higher robustness to a change in quality heterogeneity for the result of BiGbottom and MAIC, the most likely reason is that they explicitly model and estimate the list quality. Usually, the quality and heterogeneity of quality for input datasets are hard to know, whereas the list type (ranked or unranked) and the number of sources are relatively obvious. So, among top-performing methods in the evaluation of this study, methods like BiGbottom and MAIC that parameterize list quality and tend to be robust for various quality heterogeneity are preferred if the heterogeneity is unknown.

General noise level is another property that is usually hard to know in real data. The selected best-performing methods for each dataset type and heterogeneity level shown in Figure 3 are robust to various noise levels under the corresponding scenarios where they give the best performance. Similarly, these selected best-performing methods are also robust to the change of the absent gene rate (*γ*) for sources and the cutoffs for the results (the number of top-ranked genes in the result list used to calculate the accuracy) when calculating the accuracy, shown in Supplementary File S1—Figure S6, with slight fluctuations of their rankings under their best-performed dataset types and heterogeneity. Comparison between top-100 accuracy, which is also plotted in Supplementary File S1—Section S4, and top-1000 accuracy can also suggest the robustness on cutoffs of the results used to evaluate the performance for each method. It can be noticed that the performance ranking of MAIC and BIRRA shows a significant decreasing trend when the cutoff length falls for datasets with only ranked lists and high heterogeneity, especially for small datasets, whereas BiGbottom is more robust for small cutoffs (shown in Fig. 3d). So although they appear together with BiGbottom as the top-ranked methods for ranked only data with high heterogeneity depending on the results of top-1000 cutoff, BiGbottom is preferred if a study focuses on a small group of top-ranked entities within the result. Considering the robustness of cutoffs, only BiGbottom is selected as the final best-performing method in Figure 3 where the input data only include a few ranked lists with high heterogeneity of quality. But MAIC and BIRRA can also be expected to show the best level of performance when a study focuses on more genes in the result, like top-1000 genes.

In addition to the important features of genomic data explored in previous research published by Li *et al.* (2019) which also compared ranking aggregation methods on genes, this study proposed a simulated data generator after analysing three collected real datasets to systematically include key features neglected by previous researches which can influence the result significantly, including the inclusion of unranked lists, various heterogeneity of quality, the large numbers of genes in real cases (20 000), the distribution of list length, various size of lists and relationship between each gene in the setting of ground truth. Among these features, dataset size, the inclusion of unranked lists and heterogeneity which vary from datasets can usually be available for the user of ranking aggregation algorithms to some extent and can influence the performance of the algorithms significantly, as shown in the evaluation results of this study. The result of this study is summarized as a flowchart to guide the choice of ranking aggregation methods depending on these features. Advanced methods recently proposed (MAIC and BiG) which are not included by Li *et al.* (2019) are included in this study and they show top-ranked performance in many scenarios.

In this study, datasets with only order information of some entities are evaluated, including the ranked or unranked list of genes. But methods like MAIC and BiG can also take the classification of sources as additional input information. Classification labels can be manually assigned to each source by classifying sources using self-defined criteria like experiment methods or cell types used. So it could be additional information that can be relatively easy to provide. In the future, the influence of classification on ranking aggregation methods and methods for classifying sources will be explored.

## Funding

This work was supported in part by funds from the MRC SHIELD consortium (DHD PI) investigating novel host based antimicrobial responses to antimicrobial resistant bacteria [MR/N02995X/1]. Edinburgh Global Research Scholarship from the University of Edinburgh to B.W.; Institute Strategic funding provided to the Roslin Institute by the BBSRC [BBS/E/D/10002070 and BBS/E/D/10002071 to A.L.]. JKB gratefully acknowledges funding support from a Wellcome Trust Senior Research Fellowship (223164/Z/21/Z), UKRI grants MC_PC_20004, MC_PC_19025, MC_PC_1905, MRNO2995X/1, and MC_PC_20029, Sepsis Research (Fiona Elizabeth Agnew Trust), a BBSRC Institute Strategic Programme Grant to the Roslin Institute (BB/P013732/1, BB/P013759/1), and the UK Intensive Care Society. For the purpose of open access, the author has applied a CC BY public copyright licence to any Author Accepted Manuscript version arising from this submission.

*Conflict of Interest*: The authors have no financial interests to declare. Since our intention is to fairly evaluate a range of methods, it is relevant that J. Kenneth Baillie conceived the MAIC algorithm and Bo Wang, Andy Law and Michael U. Gutmann have worked extensively on development and optimisation of MAIC.

## Supplementary Material

btac621_Supplementary_Data

## Data Availability

Supporting data 1–7 (supporting results and collected real genomic data) are available on GitHub at: https://github.com/baillielab/comparison_of_RA_methods.
