# Supplementary material for: Systematic comparison of ranking aggregation methods for gene lists in experimental results
Source: Bioinformatics. 2022 Sep 12;38(21):4927–33. doi: 10.1093/bioinformatics/btac621 (PMC9620830; doi:10.1093/bioinformatics/btac621)
Supplement: btac621_Supplementary_Data [file btac621_supplementary_data.pdf]

Supplementary file 1

This is Supplementary file 1, whereas Supporting data 1-7 and the code used in this study are available on GitHub, at: `https://github.com/baillielab/comparison_of_RA_methods`

Supplementary file 1: The description for real data collection, the exploration for parameter settings of the stochastic generative model, the selection and implementation details for investigated ranking aggregation methods and some result figures of the evaluation.

Supporting data 1: "1_accuracy_M_D_initial_explore.csv" Result table for exploring simulation parameters about M (mean noise) and D (heterogeneity).

Supporting data 2: "2_accuracy-C_plot_D_gamma_average.csv" Result for evaluation on simulated data with various cutoffs of result and absent gene rate γ, plotted as Figure 6 in Supplementary file 1. The mean value of 100 repeated experiments is recorded.

Supporting data 3: "3_top-100 accuracy-M_plot_D_all.csv" Result for evaluation on simulated data for 18 methods and their typical variations. Accuracy with 100 cutoff for the evaluation of datasets including $M \in \{1, 3, 4\}$ for $D \in \{0.1, 0.5, 1, 3, 12\}$. Results for 100 repeated experiments are included.

Supporting data 4: "4_top-100 accuracy-D_plot_M_all.csv" Result for evaluation on simulated data for 18 methods and their typical variations. Accuracy with 100 cutoff for the evaluation of datasets including $D \in \{0.1, 0.5, 1, 3\}$ for $M \in \{0.5, 1, 3, 4, 12\}$. Results for 100 repeated experiments are included.

Supporting data 5: "5_top-1000 accuracy-M_plot_D_all.csv" Result for evaluation on simulated data for 18 methods and their typical variations. Accuracy with 1000 cutoff for the evaluation of datasets including $M \in \{1, 3, 4\}$ for $D \in \{0.1, 0.5, 1, 3, 12\}$. Results for 100 repeated experiments are included.

Supporting data 6: "6_top-1000 accuracy-D_plot_M_all.csv" Result for evaluation on simulated data for 18 methods and their typical variations. Accuracy with 1000 cutoff for the evaluation of datasets including $D \in \{0.1, 0.5, 1, 3\}$ for $M \in \{0.5, 1, 3, 4, 12\}$. Results for 100 repeated experiments are included.

Supporting data 7: "7_encoded_lists.zip" Encoded collected real lists and the encoded gold standard used in the evaluation.

1 Real Data Collection

As shown in the Table 1 of the main document, three real datasets are collected, corresponding to viral infection (SARS-CoV-2), bacterial infection (macrophages

apoptosis) and cancer (NSCLC).

The virus data comes from a SARS-CoV-2 related meta-analysis study for prioritisation of host genes implicated in COVID-19 [Parkinson et al., 2020]. The dataset used in the evaluation to assess the result is selected from the lists collected by researchers later than the first published version (2020-07-9 to 2020-11-25) and is not used as input for the ranking aggregation algorithms. 238 genes appearing more than once within the top 100 genes of new lists after the published lists (2020-07-9) are extracted as truth. Details of each source for SARS-CoV-2 data can be found in [Parkinson et al., 2020].

The cancer dataset consists of genes associated with non-small cell lung cancer (NSCLC) which were collected and used for evaluating ranking aggregation methods by Li et al. (2019, 2018) [Li et al., 2019, 2018]. 5 lists were extracted from sources that were used in these two papers. The evaluation list used as the truth set in [Li et al., 2018] is used to assess the performance of the ranking aggregation methods.

The third dataset concerns genes related to apoptosis in human macrophages during bacterial infection. Two of the lists are provided by the SHIELD Consortium (SHIELD AMR Research Consortium: `https://shieldamrresearch.org/`) including a ranked list from an infection-associated apoptosis CRISPR screen in human iPSC-derived macrophages and another ranked list of human orthologues of upregulated genes in murine bronchoalveolar lavage alveolar macrophages (BAL AM) 16 hours after infection with Streptococcus pneumoniae [Preston et al., 2019]. Whereas the other lists for this macrophage apoptosis data set are collected from published papers including [Huang et al., 2020, Kumar et al., 2010, MacHugh et al., 2012, Yeung et al., 2019, Abebe et al., 2010, Losick and Isberg, 2006, Maertzdorf et al., 2011, Lai et al., 2020].

The dataset used as the gold standard to assess the performance of the methods is extracted from multiple sources which are either more highly related to the research question of the corresponding meta-analysis or published more recently. It is the same case for all three real datasets. So these sources used as the gold standard are assumed to be relatively reliable and combined as an unranked list for each of the three collected real dataset to assess the performance of the algorithms.

2 Parameter settings for the stochastic generative model and comparison with real data

Datasets for SARS-CoV-2, NSCLC, and macrophage apoptosis are used for exploring the features of real data, so that parameters of the stochastic generative model can be selected to better emulate the real data. After the analysis of real data shown as below, parameters are selected for generating simulated data such that the simulated data well emulates them, shown in Tables 1, 2 and 3. List number, order information, list length, frequency decay of top-ranked entities, list quality and absent genes were assessed.

In the name of dataset types (S), "r" means it only includes ranked sources whereas "m" refers to a mix of ranked and unranked sources. In addition, "0" and "1" means large dataset and small dataset respectively. By analysing the mean and standard deviation of the accuracy of real sources, $M \in \{0.5, 1, 3, 4, 12\}$ and $D \in \{0.1, 0.5, 1, 3, 12\}$ were used in the evaluation for each of the 4 dataset types to emulate various noise levels and heterogeneity. Various absent gene rates (γ) were also explored.

Table 1: Table for parameter used in simulated data generation.

list group name	number of lists	$\ln(m_c)$	D_c	M	D	γ	μ_k, k=1,...,1000	L	U
$D1_{ranked}$	11 ranked lists	5.3597	1.8055	{0.5, 1, 3, 4, 12}	{0.1, 0.5, 1,3, 12}	{0, 0.2, 0.5} when M=3, 0 for others.	$\mu_k \propto a * \exp(b * k) + c$ a=0.1405 b=-0.0099 c=0.0625, $\mu_1 = 2$	2	20000
$D1_{unranked}$	21 unranked lists	3.4365	1.6937						
$D2_{ranked}$	5 ranked lists	7.4141	1.8634						
$D2_{unranked}$	5 unranked lists	2.9905	1.3390						

Table 2: Table for the component of each data set used in simulated data generation.

Dataset type (S)	Component	Description	Emulated real dataset
0r	$D1_{ranked}$	RankLarge	large dataset: SARS-CoV-2
0m	$D1_{ranked} + D1_{unranked}$	MixLarge	
1r	$D2_{ranked}$	RankSmall	small datasets: NSCLC, macrophage apoptosis
1m	$D2_{ranked} + D2_{unranked}$	MixSmall	

The key properties for each type of simulated datasets are introduced. For example, a dataset of m0 type includes a group of $D1_{ranked}$ lists and a group of $D1_{unranked}$ lists, with a description "MixLarge" in the main document. The corresponding emulated real dataset is also introduced.

Table 3: Table for the correspondence between the properties of real data and parameters of the generative model.

**Real data**	list length boundary	list length distribution	frequency decay of top-ranked entities	list quality	absent genes
**Simulation**	L, U	m_c, D_c	μ_k	σ_i,M, D	γ

The parameters were set to emulate properties of the real data.

List number: The number of lists used in the SARS-CoV-2 study is 32, which includes 21 unranked lists and 11 ranked lists. The small data set used for NSCLC has 5 ranked lists whereas 4 ranked lists are collected for macrophages apoptosis with another 2 unranked lists. Because some unranked sources are used for extracting the gold standard for NSCLC and macrophage apoptosis data, the total number of unranked sources which can be used as input should be higher if collecting data in a similar way, although these 2 datasets are not all sources from a thorough review for related studies. For the simulated dataset, 4 scenarios are analysed. The first two are formal biological meta-analysis analysis cases emulating the collected SARS-CoV-2 data, with 11 ranked lists with or without 21 unranked lists. The other two cases emulate the small collected dataset with 5 ranked lists, and with or without 5 unranked lists.

List length: The length of each list can go from 1 to 20000 (human genome scale) theoretically. But studies that included a single gene were excluded in the SARS-CoV-2 study. Also, methods like MAIC are designed for accepting lists with at least 2 entities. So we set a boundary $L = 2$ as the least number of entities in a list. Besides, considering the human genome scale, 20000 is assigned to U as the upper bound to show all generated lists longer than U as a human genome-wide source with 20000 genes.

Length distribution: For each list in the study of SARS-CoV-2 with length denoted by h, the mean and standard deviation for $\ln(h-2)$ are calculated. The mean for ranked lists is 5.3597 and the standard deviation is 1.8055. So $\ln(m_c)$ and D_c are set to be 5.3597 and 1.8055 separately. The calculated $\ln(m_c)$ and D_c for unranked data lists are 3.4365 and 1.6937, which are the corresponding mean and standard deviation for $\ln(h-2)$. The ranked lists and unranked lists are analysed separately because unranked lists tend to be shorter. Because of the lack of lists for NSCLC and macrophage apoptosis data, especially for the unranked lists in terms of NSCLC data, they are both considered as small datasets and analysed together. The histogram and mean value of $\ln(h-2)$ for real data are shown in Figure 1, whereas selected parameters for $\ln(m_c)$ and D_c are shown in Table 1.

Entity significance μ_k: In the simulated data generation model, 0 is used as the μ_k for all noise entities. But the relationship between μ_k for signal entities needs to be explored.

The frequency of each gene that appears in the top 1000 of all sources was calculated independently for 3 real datasets. For SARS-CoV-2 data, 986 out of 11470 entities show a frequency larger than 0.05 and are considered as signal entities whereas the others are considered as noise. An exponential curve $y_k = a * \exp(b * k) + c$ is fitted for the signal entities ranked by the frequency, with rank k and frequency y_k. The $y_k = a * \exp(b * k) + c$ value with these fitted parameters can be used to estimate the significance of an entity(μ_k). The same analysis is carried out for NSCLC and macrophage apoptosis data, shown in Figure 2. As the bottom right of the figure shows, the curves for 3 real data sets are scaled to set the first value to be 2 since only the ratio between different positions is investigated here. These curves are similar, all falling from quickly to slower and tending to be flat reaching the 1000th position. So an assumption is made that all these data have a close pattern of significance falling along with the signal genes. The scaled value y_k of the curve for SARS-CoV-2, which is fitted using the most number of lists, is used to set the μ_k value, which goes from for μ_1 to for μ_{1000}. The fitted curves for the simulated data with $M = 3$ and $D \in \{1, 0.5, 0.1\}$ (explored to simulate appropriate mean noise and variance for simulated data described in the following part) are compared with the curves for 3 real data sets after scaling.

List quality σ_i: Quality of lists in real data was compared to simulated data with various M and D, to explore the appropriate settings for parameters M and D which were shown in Table 1. The genes within the corresponding extracted unranked gene set used to assess the performance of algorithms are considered as truth, with n_T genes. In order to assess the quality of each list,

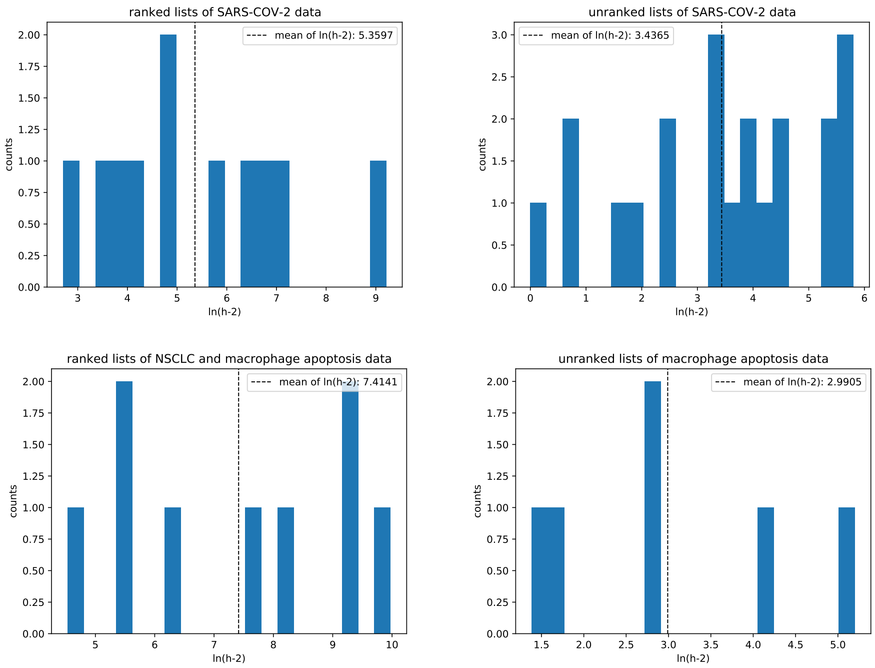

Figure 1: The histogram of $\ln(h-2)$ for SARS-CoV-2 data, and the combination of NSCLC and macrophage apoptosis data, shown as counts and mean value. Figures above are SARS-CoV-2 data. The left bottom one is the analysis of the combination of NSCLC and macrophage data, whereas the right bottom one is for all collected unranked data for macrophage apoptosis, including the constituent of extracted truth.

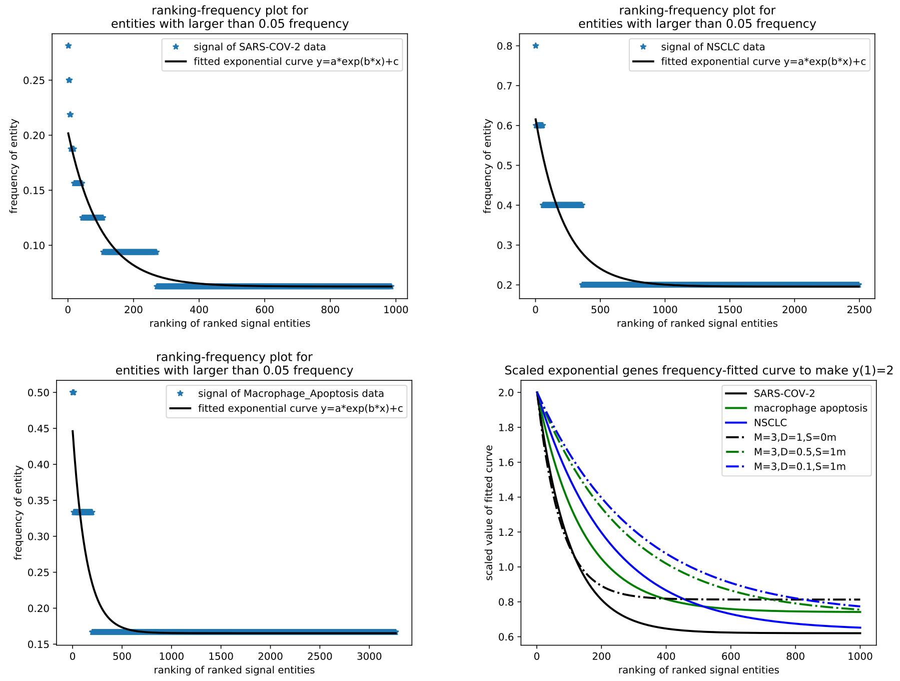

Figure 2: The left and top 3 figures show the frequency for each entity appearing in top 1000 of each list from each real data set except for the pre-selected gold standard list. Entities with frequency larger than 0.05 are considered as signals and ranked by the corresponding frequency. A curve is fitted for each dataset. The right bottom figure shows the plotting for these scaled curves with top ranked entity having value 2, together with the analysis for selected simulated data.

Table 4: Table for the calculated accuracy for NSCLC, macrophage apoptosis, and SARS-CoV-2 datasets independently.

Dataset	Length of gold standard set	Accuracy		Upper bound estimation	Lower bound estimation
		mean	standard deviation	mean accuracy	mean accuracy
SARS-CoV-2	238	0.1053	0.1222	0.2993	0.0117
NSCLC	148	0.0283	0.0263	0.3019	0.0074
macrophage apoptosis	91	0.0512	0.0951	0.2124	0.0045

For each real dataset, the length of gold standard set, calculated accuracy, upper bound and lower bound estimation are introduced.

the precision($\frac{TruePositive}{PredictedPositive}$) for each list is calculated, which is the ratio for the number of true genes appearing in top n_T genes of a ranked list with more than n_T genes. For an unranked list with length h larger than n_T, n_T genes are randomly selected from the list as the predicted positive since there is no order information provided between genes within an unranked list. For a list with h genes less than n_T, the precision value is the ratio of true genes appearing in the list. The quality of lists in real data was explored, shown as Table 4. This precision value shows the quality of each list, which is related to the σ_i in the simulated data generation model. The average of these precision values is 0.0283, 0.0512 and 0.1053 for NSCLC, macrophage apoptosis, and SARS-CoV-2 respectively. The standard deviations (std) for each of them are 0.0263, 0.0951, and 0.1222. The number of gold standards set N_T for NSCLC, macrophage apoptosis, and SARS-CoV-2 are 148, 91 and 238 separately. Because the size of the truth set may influence the accuracy, these numbers are analysed separately. The unranked lists used as the true positive for these real data are extracted from multiple sources as estimations for the corresponding truth. This estimation can influence the precision values calculated a lot if it is far from the truth. An estimation of the upper bound under various true positive sets with the same length (N_T) can be calculated by using the most common positive reported genes as the truth, which are 0.3019, 0.2124, and 0.2993 separately as shown in Table 4. The expected precision value for a non-information random selected list from 20000 genes are $N_T/20000 = 0.0074, 0.0045, 0.0117$ as shown in Table 4, which can be an estimation for the lower bound of precision values.

To explore a suitable noise level M and heterogeneity D used in the simulated data generation model, experiments on various M and D were made to compare with the real data, with other parameters set as Table 1 for a large dataset $m0$ (11 ranked + 21 unranked) and a small dataset $m1$ (5 ranked + 5 unranked) as shown in Table 2. $\gamma = 0$ is used here. $M \in \{0.5, 1, 2, 3, 4, 12\}$ is explored when $D \in \{0.1, 0.5, 1, 3, 12\}$. The no-heterogeneity scenario that M is constant is also explored, shown as $D = 0$. These M value are selected to show various signal noise ratio: $\frac{\mu_k}{\sigma_i}$. Considering the expectation $\mathbb{E}[\ln(\sigma_i)] = \ln(M)$, $\frac{\mu_k}{M}$ is used to show the estimated scale for signal-to-noise ratio(SNR). Since $\mu_k \in [0.6202, 2]$ when k=1,...1000, 1 is selected for μ_k to calculate this figure. $SNR \in \{2, 1, 0.5, 0.333, 0.25\}$. Experiments are repeated 10 times and the average of results are calculated for both mean accuracy and standard deviation of accuracy, shown as Figure 3 and Figure 4. The details are shown in Supporting data 1 as a table. The result shows M=3, D=1 best emulates the SARS-CoV-2 data by

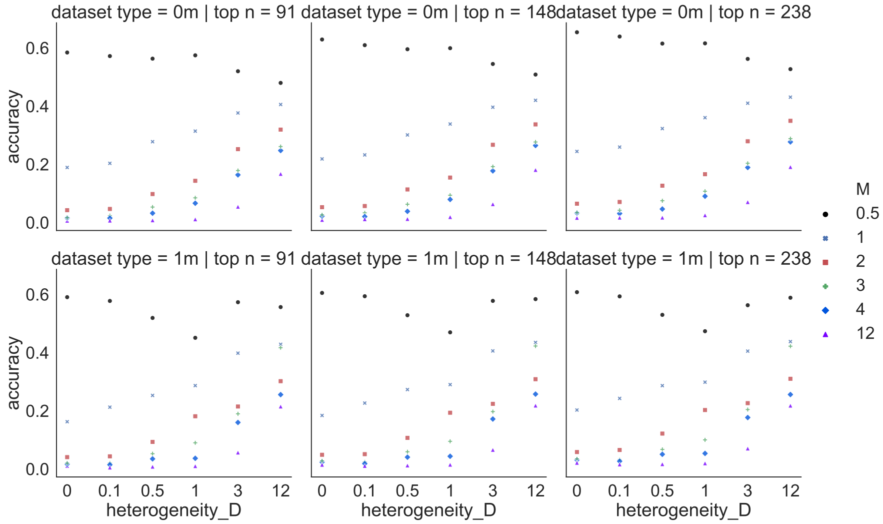

Figure 3: The figure shows the plot for mean accuracy calculated under $M \in \{0.5, 1, 2, 3, 4, 12\}$ and $D \in \{0, 0.1, 0.5, 1, 3, 12\}$. $D = 0$ means M is constant. Each result is an average of 10 repeat experiments.

generating lists with a mean top-238 accuracy of 0.1073 and standard deviation of 0.1858 for the large dataset. For the top-148 accuracy, the mean and std value for NSCLC are 0.0283 and 0.0263. Simulated data with M=3 and D=0.1 has similar values with 0.0245 mean and 0.0275 std value for the small dataset. In terms of the top 91-accuracy, the mean and std value for macrophage apoptosis are 0.0512 and 0.0951. Data with M=3 and D=0.5 has similar values with 0.0525 mean and 0.0694 std value for the small dataset. So M=3 will be treated as a classic important setting when exploring other parameters.

Estimated lower bounds and upper bounds are also compared to explore parameters for emulating a large enough range of various real cases. The smallest mean values for accuracy are around 0.0106, 0.005, and 0.017 for M=12, D=0.1 with the corresponding accuracy for top-N_T cutoff, which are close to the non-information list value (the lower bound estimation) mentioned above and low enough for emulating the lists in real data. It goes to around 0.6 when $M = 0.5$, which is far higher than the upper bound estimation for 3 real datasets and larger than any collected real list accuracy, which tends to be high enough to emulate the real data. In terms of the range of the heterogeneity, the std of accuracy goes from close to no heterogeneity ($D = 0$) when $D = 0.1$ to around 0.4 when D is larger, which is times higher than any real calculated std and seems high enough in the real case.

Considering the analysis above, parameters settings for M and D used in the evaluation are selected. Various M and D will be used to explore

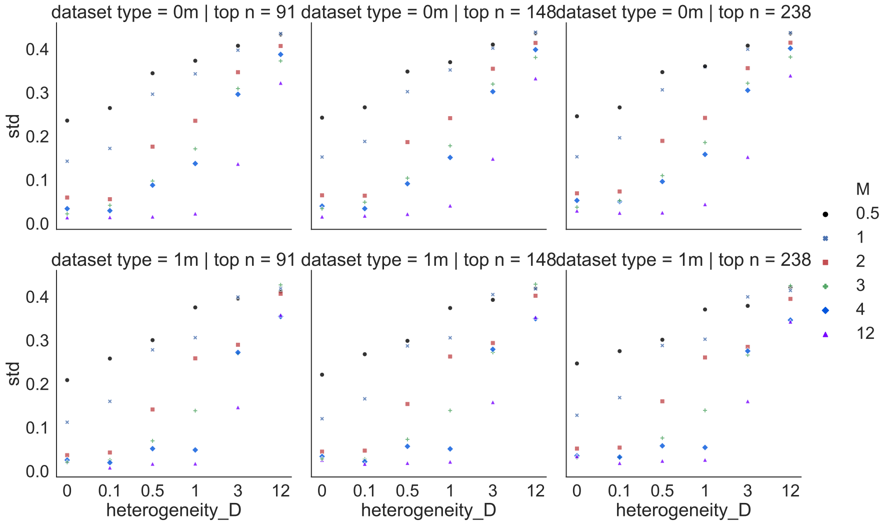

Figure 4: The figure shows the plot for standard deviation of accuracy, calculated under $M \in \{0.5, 1, 2, 3, 4, 12\}$ and $D \in \{0, 0.1, 0.5, 1, 3, 12\}$.$D = 0$ means M is constant. Each result is an average of 10 repeat experiments.

themselves besides $M = 3$ and $D \in \{0.1, 0.5, 1\}$. $M \in \{0.5, 1, 3, 4, 12\}$ and $D \in \{0.1, 0.5, 1, 3, 12\}$ are used to explore various average quality and the heterogeneity of list quality. For heterogeneity D, it is the variance of $\ln(\sigma_i)$ with expectation $\mathbb{E}[\ln(\sigma_i)] = \ln(M)$ in the data generation model. When $M = 3$, $(ln(M))^2 = 1.2$. The corresponding signal-to-noise ratio $SNR = \frac{(\ln(M))^2}{D} \in \{12, 2.4, 1.2, 0.4, 0.1\}$ for $D \in \{0.1, 0.5, 1, 3, 12\}$. It can also support that the settings for M and D can generate datasets to cover cases of various mean noise and heterogeneity including common cases in real data for a large reasonable range of SNR for both $\frac{\mu_k}{M}$ and $\frac{(\ln(M))^2}{D}$.

Absent genes γ: For the absence of genes in a real study, it is usually hard to judge the cases between bottom omitted or randomly excluded because the genes included in a study are usually more or less selected using prior knowledge. A simple way to emulate the combination of various scenarios for the exclusion of genes is used by randomly removing 20% and 50% of genes to be removed before cutting the bottom genes for $M = 3$, $D \in \{0.1, 0.5, 1, 3, 12\}$ in terms of 4 types of datasets in Table 2.

3 Methods selection and implementation

As shown in Section 2.3 of the manuscript, the ranking aggregation methods with various features which can deal with gene lists were selected and implemented, with the settings summarised in Table 5. For each method, the table introduces

Table 5: Table for the ranking aggregation methods investigated in this study.

Method	Code Source if used	New implementation	Parameter Settings	Unranked List Used
MAIC	[Li et al., 2020], publicly available	no	Default, source entities number=20000	True
VC		yes	Default	True
RRA	R package:RobustRankAggreg	no	source entities number=20000	False
RepeatChoice		yes	default	True
BIRRA	[Badgeley et al., 2015], publicly available	no	Default	False
rMED, rMEAN, rGEO	R package:RobustRankAggreg	no	source entities number=20000	False
Stuart	R package:RobustRankAggreg	no	source entities number=20000	False
tMED, tMEAN, tGEO, tL2	R package:TopKLists	no	Default	False
rMixMED, rMixMEAN, rMixGEO	R package:RobustRankAggreg	edited to include unranked list	source entities number=20000<br>unranked entities = 1/2 length	True
MixStuart	R package:RobustRankAggreg	edited to include unranked list	source entities number=20000<br>unranked entities = 1/2 length	True
BiGbottom	[Li et al., 2018], provided by the authors	no	2000 iterations, 1000 burnin, no platform,<br>genes not in list are treated as bottom ranked	False
BiGNA	[Li et al., 2018], provided by the authors	no	2000 iterations, 1000 burnin, no platform,<br>genes not in list are treated as unknown	False
MC1-3	R package:TopKLists	no	Default	False
BARD	[Deng et al., 2014], provided by the authors	no	Default	False

the method name, code sources if used, the new implementation in this study besides enabling them to deal with data in the same format, parameter settings, and if using unranked lists.

The earliest methods use simple statistics and Borda's methods are the most popular cases [de Borda, 1781, Lin, 2010]. This type of methods calculates statistics like the mean rank of each entity to get the aggregated result rank. VoteCounting used in [Li et al., 2020] also uses simple statistics, which are easy to implement. Bayesian methods are becoming more popular these years [Badgeley et al., 2015, Deng et al., 2014, Yi et al., 2016, Li et al., 2018]. BIRRA [Badgeley et al., 2015] initially marks a group of entities as truth and repetitively updates this set. BARD [Deng et al., 2014] defines a model with an independent parameter for each list to control the probability of position for true entities among noise entities within the corresponding list. In contrast, BiG [Li et al., 2018] and BARC [Yi et al., 2016] use a Thurstone-based model [Thurstone, 1927], which assumes there is a parameter for each entity and each ranking source is ranked by these parameters subject to noise. Given the solid Bayesian theoretical framework provided, the results can usually be shown in form of an estimated distribution instead of only a single ranking. But it often provides a result largely influenced by priors which are manually defined by the user or specific method. The computational cost is also likely to be large since Monte Carlo methods are usually used to estimate the posterior. BARD and BiG methods are more time-consuming than other methods except for Markov chain methods (MC1-3) that can also take days to run for a large dataset. In order to complete each running within or around a reasonable time (48 hours), the same omitting method is used as in [Li et al., 2018] and [Li et al., 2019]. Before running BARD, BiG (BiGbottom and BiGNA) or MC1-3 on a dataset, genes not ranked within the top 1000 in any list are removed because they are less likely to be ranked within the top 500 in the final result. After this operation, the number of entities in the SARS-CoV-2, NSCLC, and macrophage apoptosis data set became 4584, 2495, and 3267 respectively. The maximal number of iterations in BiG algorithms is set to be 2000 to reduce the running time. Figure 5 shows the comparison between the result of using 5000 iterations and 2000 iterations for BiGbottom (BiG treating absent genes as bottom-ranked) and BiGNA (BiG treating the

order information of absent genes as unknown). They tend to show the same result so that reducing the number of iterations is not likely to influence the accuracy of the BiG algorithm much and is hence reasonable given the reduction in computing time. Because category information is not investigated in this study, a no-platform version of BiG is applied for both BiGbottom and BiGNA. Markov chain methods set each unique entity within all input lists as a state and rank entities by the probability of their stationary distributions [Dwork et al., 2001].

In addition to the Bayesian and Markov chain methods, there are other methods that also use the distribution of input rankings or propose a model with distributions like Normal distributions to model the noise which causes the variability within a list, like Stuart [Stuart et al., 2003] and Robust Ranking Aggregation(RRA) [Kolde et al., 2012]. The R Package RobustRankAggreg allows the user to set the number of entities and this parameter in all related implementations is set to be 20000 to emulate the human genome scale, including RRA, Stuart, rMED, rMEAN, rGEO, and the Mix version of Stuart and Borda's methods. These 'Mix' versions of Borda's methods work exactly the same as the original implementations in the RobustRankAggreg package when dealing with a dataset with ranked sources only

MAIC also allows setting the number of entities, which was again set to 20000. Considering the variation of source quality, some methods try to recognise the difference between lists by weighting them using pre-defined weighting. Pre-defined weighting schemes like in CEMC [Lin and Ding, 2009] require information in addition to the order information, which is usually not available. Methods like MAIC [Li et al., 2020] define latent variables to weight the quality of each source and estimate them without additional information.

RepeatChoice [Ailon, 2010] is also investigated as a method which can deal with a mix of ranked and unranked data and includes stochastic processes.

4 Supplementary result figures

This section shows the supplementary plotting for results. Figure 5 shows parameter setting exploration for BiGbottom and BiGNA algorithms, by setting iterations to be 2000 and 5000. Similar results are shown by these two settings for each algorithm in three real datasets, which supports the selection of iterations for BiG algorithm. Figure 6 to Figure 12 shows the supplementary results for the comparison of ranking aggregation methods on simulated and real datasets. Figure 7 shows the top-1000 cutoff results of experiments for 3 real datasets and selected simulated datasets with various key features, including the number of included lists, whether unranked lists are included and the heterogeneity of list quality. To avoid duplicates, the 'Mix' version of Borda's methods and Stuart are only shown in the result of datasets with unranked sources since they perform exactly the same as the corresponding original version in the R Package RobustRankAggreg for ranked sources. It can be noticed that the ranking of performance for investigated methods is influenced by the datasets. For example,

the performance of BIRRA is the best among the investigated methods for the large dataset with only ranked sources and high heterogeneity (RankLarge highHet), whereas it is not the best-performed method in the corresponding datasets with low heterogeneity (RankLarge lowHet) or with a mix of ranked and unranked sources (MixLarge highHet). Another example can be that rMixGEO has the higher ranking of the performance when heterogeneity is high for the simulated datasets.

The significant influence on the performance of ranking aggregation methods caused by the investigated features means that knowing the dataset features can help select appropriate methods to achieve better performance. A detailed analysis about the relationship between dataset features and the performance of ranking aggregation methods is provided in the Result and Discussion Section of the main document.

Parameter setting exploration for BiG

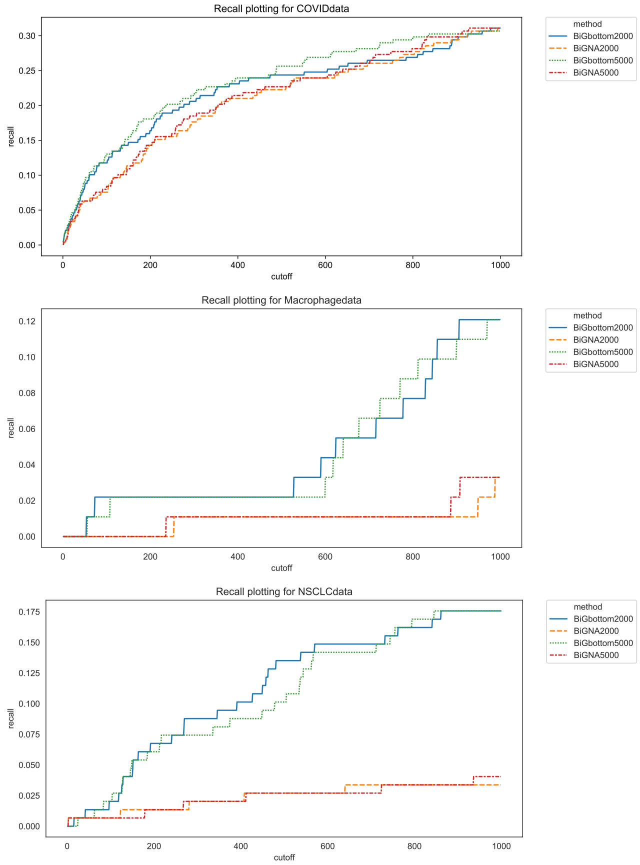

Figure 5: Parameter setting exploration for BiGbottom and BiGNA. The recall for top 1 to 1000 genes in the result list of 3 real datasets for BiGbottom and BiGNA, running with 2000 iterations and 5000 iterations.

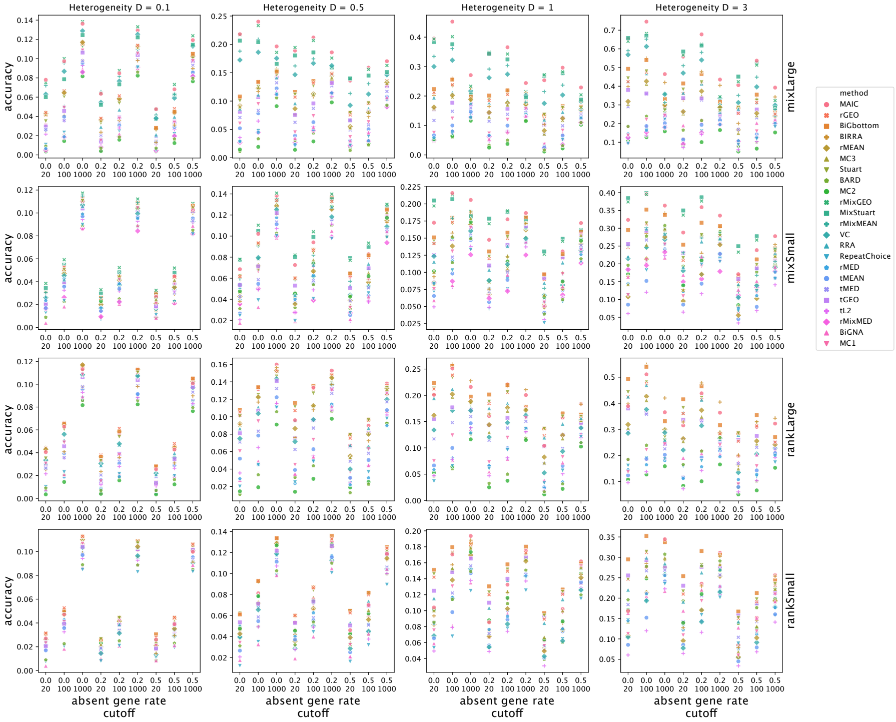

Figure 6: Result for simulated datasets with various absent gene rates and cutoffs. Average value of 100 replicates for each setting is plotted. The accuracy for top 20, 100, and 1000 genes in the result list of simulated data sets are plotted with the change of gene absent rates ($\gamma \in \{0, 0.2, 0.5\}$) when the quality heterogeneity goes from really low to high ($D \in \{0.1, 0.5, 1, 3\}$) and mean noise level emulates the default classic scenario ($M = 3$). Simulated dataset types are shown as {MixLarge, MixSmall, RankLarge, RankSmall} corresponding to $S \in \{0m, 1m, 0r, 1r\}$ to show whether unranked lists are included and the number of included lists.

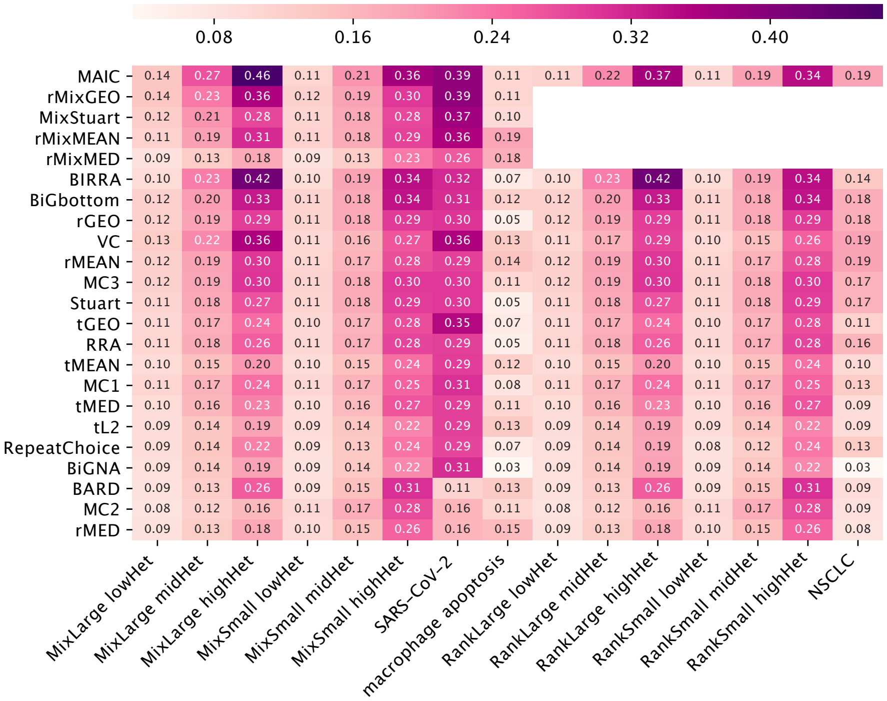

Figure 7: Coverage rates with top-1000 cutoff for important experiments. The name of each investigated method is shown along y-axis. For simulated data, it includes top-1000 accuracy for scenarios with the default mean noise level $M = 3$ and absent gene rate $\gamma = 0$. The dataset label for simulated data shown along x-axis is the combination of dataset type and heterogeneity. Simulated dataset types are shown as {MixLarge, MixSmall, RankLarge, RankSmall} corresponding to $S \in \{0m, 1m, 0r, 1r\}$ to show whether unranked lists are included and the number of included lists. The quality heterogeneity is recorded as lowHet, midHet and highHet, corresponding to the small quality heterogeneity $D = 0.1$, the medium one $D = 1$ and the large one $D = 3$ separately. For each simulated data setting, the mean value of the results from 100 repeated experiments is plotted. Top-1000 recall for 3 experiments using real datasets(SARS-CoV-2, macrophage apoptosis and NSCLC) are also included.

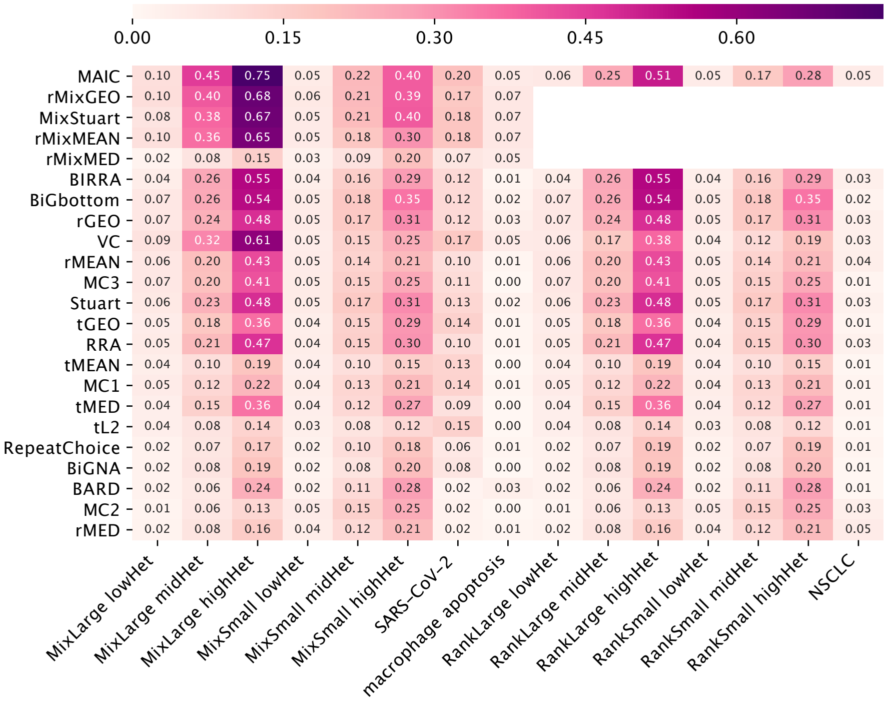

Figure 8: Coverage rates with top-100 cutoff for important experiments. The name of each investigated method is shown along y-axis. For simulated data, it shows top-100 accuracy for scenarios with the default mean noise level $M = 3$ and absent gene rate $\gamma = 0$. The dataset label for simulated data shown along x-axis is the combination of dataset type and heterogeneity. Simulated dataset types are shown as {MixLarge, MixSmall, RankLarge, RankSmall} corresponding to $S \in \{0m, 1m, 0r, 1r\}$ to show whether unranked lists are included and the number of included lists. The quality heterogeneity is recorded as lowHet, midHet and highHet, corresponding to the small quality heterogeneity $D = 0.1$, the medium one $D = 1$ and the large one $D = 3$ separately. For each simulated data setting, the mean value of the results from 100 repeated experiments is plotted. Top-100 recall for 3 experiments using real datasets(SARS-CoV-2, macrophage apoptosis and NSCLC) are also included.

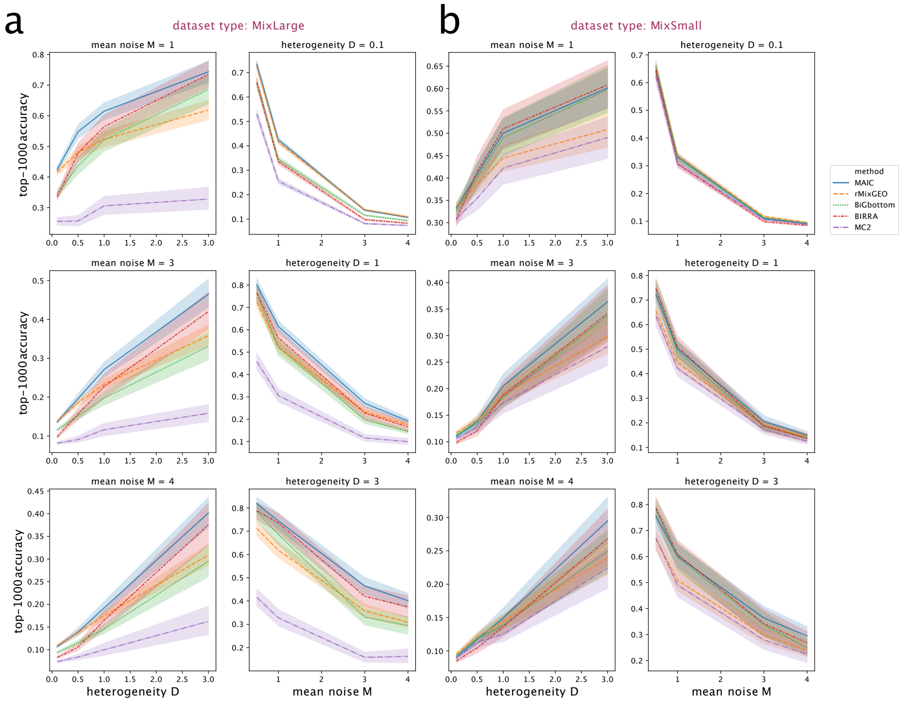

Figure 9: Results (top-1000 cutoff) for simulated datasets with a mix of ranked and unranked sources. All subplots use the same color and line styles to show investigated methods. (**a**) Simulated data - MixLarge: results for simulated data with various mean noise levels and quality heterogeneity. The simulated dataset type is MixLarge, corresponding to $S = 0m$ to show that the number of included lists is large. (**b**) Simulated data - MixSmall: results for simulated data with dataset type to be MixSmall, corresponding to $S = 1m$ to show that the number of included lists is small. For both (**a**) and (**b**), mean of accuracy using top-1000 cutoff and 95% confidence interval are plotted for 100 repeated experiments using lines and shading separately. The default setting of absent gene rate $\gamma = 0$ is used.

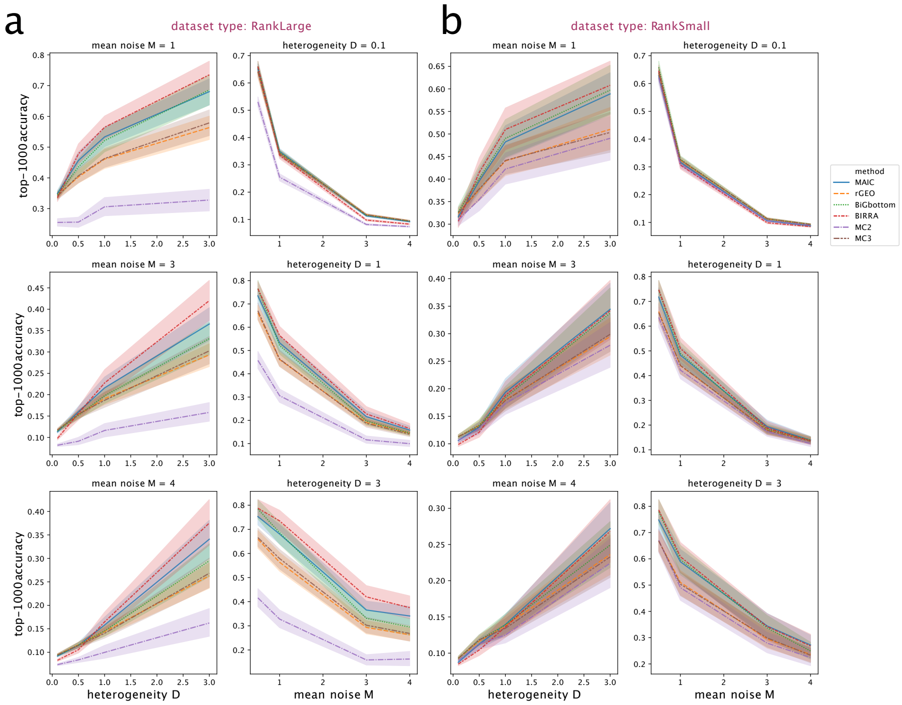

Figure 10: Result (top-1000 cutoff) for simulated datasets with only ranked sources. All subplots use the same color and line styles to show investigated methods. (**a**) Simulated data - RankLarge: results for simulated datasets with only ranked sources, investigating various mean noise levels and quality heterogeneity. The simulated dataset type is RankLarge, corresponding to $S = 0r$ to show that the the number of included lists is large. Mean of accuracy using top-1000 cutoff and 95% confidence interval are plotted for 100 repeated experiments using lines and shading separately. The default setting of absent gene rate $\gamma = 0$ is used. (**b**) Simulated data - RankSmall: Simulated dataset type is RankSmall, corresponding to $S = 1r$ to show that the number of included lists is small. Any other settings are the same as experiments shown in (**a**).

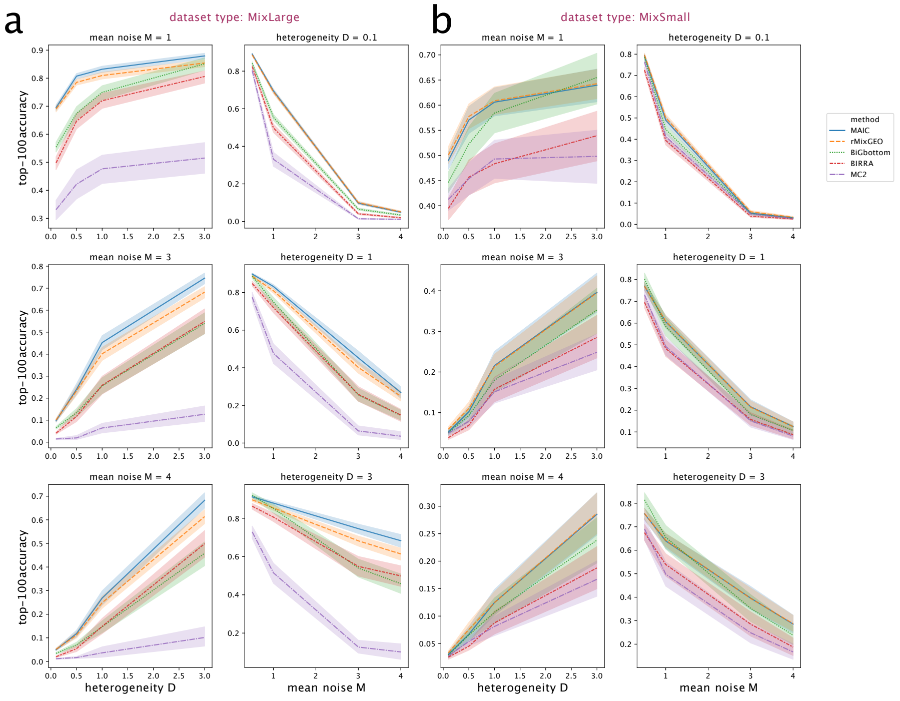

Figure 11: Results (top-100 cutoff) for a mix of ranked and unranked sources for simulated data with various mean noise level and quality heterogeneity. Mean of accuracy using top-100 cutoff and 95% confidence interval are plotted for 100 repeated experiments using lines and shadows seperately. The default setting of absent gene rate $\gamma = 0$ is used. (**a**) MixLarge: Simulated dataset types is MixLarge, corresponding to $S = 0m$ to show both ranked and unranked lists are included and the number of included lists is large for each dataset. (**b**) MixSmall: Simulated dataset types is MixSmall, corresponding to $S = 1m$ to show both ranked and unranked lists are included and the number of included lists is small for each dataset.

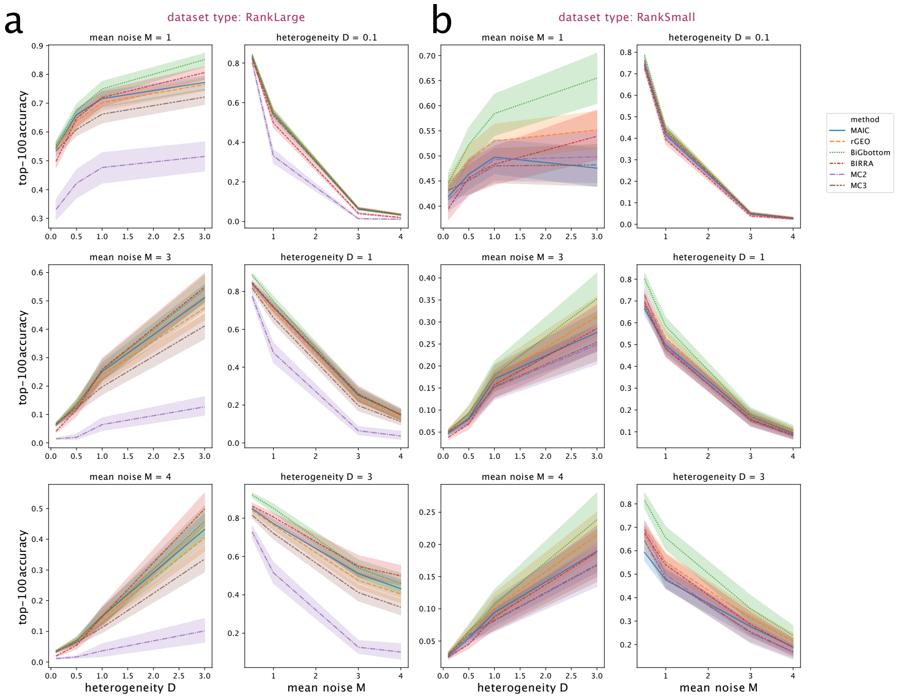

Figure 12: Results (top-100 cutoff) for simulated datasets with only ranked sources with various mean noise level and quality heterogeneity. Mean of accuracy using top-100 cutoff and 95% confidence interval are plotted for 100 repeated experiments using lines and shadows seperately. The default setting of absent gene rate $\gamma = 0$ is used. (**a**) RankLarge: Simulated dataset types is RankLarge, corresponding to $S = 0r$ to show that only ranked lists are included and the number of included lists is large for each dataset. (**b**) RankSmall: Simulated dataset types is RankSmall, corresponding to $S = 1r$ to show that only ranked lists are included and the number of included lists is small for each dataset.

References

M. Abebe, T. M. Doherty, L. Wassie, A. Aseffa, K. Bobosha, A. Demissie, M. Zewdie, H. Engers, P. Andersen, L. Kim, et al. Expression of apoptosis-related genes in an ethiopian cohort study correlates with tuberculosis clinical status. *European journal of immunology*, 40(1):291–301, 2010.

N. Ailon. Aggregation of partial rankings, p-ratings and top-m lists. *Algorithmica*, 57(2):284–300, 2010.

M. A. Badgeley, S. C. Sealfon, and M. D. Chikina. Hybrid bayesian-rank integration approach improves the predictive power of genomic dataset aggregation. *Bioinformatics*, 31(2):209–215, 2015.

J. C. de Borda. Memoire sur les elections au scrutin, 1781. *Histoire de l'Academie Royale des Sciences, Paris*, 1781.

K. Deng, S. Han, K. J. Li, and J. S. Liu. Bayesian aggregation of order-based rank data. *Journal of the American Statistical Association*, 109(507):1023–1039, 2014.

C. Dwork, R. Kumar, M. Naor, and D. Sivakumar. Rank aggregation methods for the web. In *Proceedings of the 10th international conference on World Wide Web*, pages 613–622. ACM, 2001.

T. Huang, C. Jiang, M. Yang, H. Xiao, X. Huang, L. Wu, and M. Yao. Salmonella enterica serovar typhimurium inhibits the innate immune response and promotes apoptosis in a ribosomal/trp53-dependent manner in swine neutrophils. *Veterinary Research*, 51(1):1–12, 2020.

R. Kolde, S. Laur, P. Adler, and J. Vilo. Robust rank aggregation for gene list integration and meta-analysis. *Bioinformatics*, 28(4):573–580, 2012.

D. Kumar, L. Nath, M. A. Kamal, A. Varshney, A. Jain, S. Singh, and K. V. Rao. Genome-wide analysis of the host intracellular network that regulates survival of mycobacterium tuberculosis. *Cell*, 140(5):731–743, 2010.

Y. Lai, G. H. Babunovic, L. Cui, P. C. Dedon, J. G. Doench, S. M. Fortune, and T. K. Lu. Illuminating host-mycobacterial interactions with genome-wide crispr knockout and crispri screens. *Cell systems*, 11(3):239–251, 2020.

B. Li, S. M. Clohisey, B. S. Chia, B. Wang, B. J. Kenneth, H. Nir, et al. Genome-wide crispr screen identifies host dependency factors for influenza a virus infection. *Nature communications*, 11(1):1–18, 2020.

X. Li, P. K. Choudhary, S. Biswas, and X. Wang. A bayesian latent variable approach to aggregation of partial and top-ranked lists in genomic studies. *Statistics in medicine*, 37(28):4266–4278, 2018.

X. Li, X. Wang, and G. Xiao. A comparative study of rank aggregation methods for partial and top ranked lists in genomic applications. *Briefings in bioinformatics*, 20(1):178–189, 2019.

S. Lin. Rank aggregation methods. *Wiley Interdisciplinary Reviews: Computational Statistics*, 2(5):555–570, 2010.

S. Lin and J. Ding. Integration of ranked lists via cross entropy monte carlo with applications to mrna and microrna studies. *Biometrics*, 65(1):9–18, 2009.

V. P. Losick and R. R. Isberg. Nf-κb translocation prevents host cell death after low-dose challenge by legionella pneumophila. *The Journal of experimental medicine*, 203(9):2177–2189, 2006.

D. E. MacHugh, M. Taraktsoglou, K. E. Killick, N. C. Nalpas, J. A. Browne, S. D. Park, K. Hokamp, E. Gormley, and D. A. Magee. Pan-genomic analysis of bovine monocyte-derived macrophage gene expression in response to in vitro infection with mycobacterium avium subspecies paratuberculosis. *Veterinary research*, 43(1):1–19, 2012.

J. Maertzdorf, D. Repsilber, S. K. Parida, K. Stanley, T. Roberts, G. Black, G. Walzl, and S. H. Kaufmann. Human gene expression profiles of susceptibility and resistance in tuberculosis. *Genes & Immunity*, 12(1):15–22, 2011.

N. Parkinson, N. Rodgers, M. H. Fourman, B. Wang, M. Zechner, M. C. Swets, J. E. Millar, A. Law, C. D. Russell, J. K. Baillie, et al. Dynamic data-driven meta-analysis for prioritisation of host genes implicated in covid-19. *Scientific reports*, 10(1):1–12, 2020.

J. A. Preston, M. A. Bewley, H. M. Marriott, A. McGarry Houghton, M. Mohasin, J. Jubrail, L. Morris, Y. L. Stephenson, S. Cross, D. R. Greaves, et al. Alveolar macrophage apoptosis–associated bacterial killing helps prevent murine pneumonia. *American journal of respiratory and critical care medicine*, 200 (1):84–97, 2019.

J. M. Stuart, E. Segal, D. Koller, and S. K. Kim. A gene-coexpression network for global discovery of conserved genetic modules. *science*, 302(5643):249–255, 2003.

L. L. Thurstone. A law of comparative judgment. *Psychological review*, 34(4): 273, 1927.

A. T. Yeung, Y. H. Choi, A. H. Lee, C. Hale, H. Ponstingl, D. Pickard, D. Goulding, M. Thomas, E. Gill, J. K. Kim, et al. A genome-wide knockout screen in human macrophages identified host factors modulating salmonella infection. *MBio*, 10(5), 2019.

D. Yi, X. Li, and J. S. Liu. Bayesian aggregation of rank data with covariates and heterogeneous rankers. *arXiv preprint arXiv:1607.06051*, 2016.
